# Polyampholyte Copolymers Based on Dual Ionic Monomers as Self‐Healing Aqueous Binders for Lithium Ion Battery Cathodes

**DOI:** 10.1002/cssc.202502470

**Published:** 2026-05-12

**Authors:** Jon López de Lacalle, Ana Clara Rolandi, Jorge L. Olmedo‐Martínez, Miryam Criado‐Gonzalez, Eduardo J. García‐Suárez, Nerea Casado, David Mecerreyes

**Affiliations:** ^1^ POLYMAT University of the Basque Country UPV/EHU Donostia‐San Sebastián Spain; ^2^ Center for Cooperative Research on Alternative Energies (CIC energiGUNE) Basque Research and Technology Alliance (BRTA) Vitoria‐Gasteiz Spain; ^3^ IKERBASQUE, Basque Foundation for Science Bilbao Spain

**Keywords:** battery, binder, copolymers, ionic polymer, self‐healing

## Abstract

Self‐healing polymeric binders play a key role in preserving the mechanical stability of electrodes, as they can autonomously recover from mechanical damage, such as cracks or ruptures, without affecting battery performance. Herein, a polyampholyte copolymer family is designed with tunable glass transition temperature and self‐healing properties associated with the reversible ionic and hydrogen bonding interactions. A dual ionic monomer (DIM) is synthesized by a proton transfer reaction between methacrylic acid (MA) and dimethylaminoethyl methacrylate (DMAEMA). A series of copolymers with both anionic and cationic groups in the polymer backbone were then synthesized by copolymerization of the DIM with poly(ethylene glycol) methyl ether methacrylate (PEGMEMA) at different ratios by free‐radical polymerization in water. The poly(MA‐co‐DMAEMA‐co‐PEGMEMA) random copolymers presented different glass transition temperatures (*T*_g_ ranging from −57°C to 166°C), varying from sticky polymers to very brittle membranes. The intermediate compositions, which show copolymers that are mechanically flexible and highly adhesive, were further evaluated. Self‐healing properties were investigated by optical microscopy and rheological measurements, showing intrinsic repair ability at different temperatures, such as 40°C. Finally, ionic conductivity and electrochemical battery testing of the selected polyampholyte copolymer composition corroborate the potential application as water‐soluble binders in cathodes for lithium‐ion batteries.

## Graphical abstract

In this article, water‐processable self‐healing binders for lithium‐ion batteries were developed. The polyampholyte acrylic copolymers combine ionic conductivity, tunable mechanical properties, and autonomous crack repair for lithium iron phosphate (LFP) cathode binders for lithium batteries.

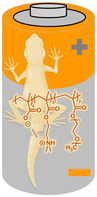

## 1 Introduction

Self‐healing polymers have recently emerged as a distinctive class of smart materials capable of autonomously repairing mechanical or microstructural damage, which enhances the durability, safety, and lifecycle of the products. From both economic and sustainability perspectives, these materials are particularly attractive, as they preserve their structural integrity and appearance across multiple damage‐healing cycles, thereby facilitating material reuse and reducing plastic waste [1, 2]. Current research is focused on the development of self‐healing systems and their potential application [4, 5] across a broad range of fields [6], including protective coatings [7, 8], biomedical devices, or energy storage systems [9, 10], sectors where premature failure can critically limit product performance.

Self‐healing polymers are classified according to their healing mechanisms, either extrinsic or intrinsic systems. Relating to the first approach, encapsulated agents such as reactive monomers or catalysts are released upon damage, enabling localized repair but often limiting the number of healing cycles. In contrast, intrinsic self‐healing is divided into reversible dynamic bonds integrated in the polymer backbone, for instance, disulfides (S─S), or in contrast, supramolecular interactions [11, 12]. Among the latter, known as noncovalent interactions, hydrogen bonding, metal‐ligand complexation, ionic interactions, and *π–* *π* stacking play a central role, enabling the formation of supramolecular polymer networks capable of repairing themselves under mild conditions, while also offering recyclability and tunable mechanical properties [13]. Ionic interactions offer many possibilities for materials design associated with their rather simple chemistry. Ionic self‐healing polymers consist of the reversible association between oppositely charged species, typically carboxylates or sulfonates as negative and metal cations or ammonium groups as positively charged ions, resulting in dynamic physical crosslinking by electrostatic forces [12, 14]. While many ionic self‐healing materials use polyampholytes [15] or charged monomers to be applied as wearable devices [16, 17], emerging systems increasingly explore neutral ionizable monomers, leading to the development of dual self‐healing ionic hydrogels [18] and elastomers [19].

As mentioned before, self‐healing polymers are being investigated to improve the safety of lithium batteries, both as polymer electrolytes or polymer binders [20, 21]. In this case, the design of the polymer aims to combine high ionic conductivity with healable mechanical properties to address the poor interfacial compatibility between electrodes, which raises safety concerns [22]. A recent review by Ahuja et al., the authors explores the advances in self‐healing electrolytes, with a focus on ionic polymers such as poly(ionic liquid)‐based materials and their structural tuning for battery performance. As a common strategy, substituting halide anions with bulkier counterparts such as sulfonamides enhances polymer chain mobility, which is essential for achieving self‐healing properties [23]. In other examples of polymer electrolytes, Elizalde et al. developed a gel electrolyte based on poly(urea‐urethane) chemistry for lithium batteries [24], and Pei et al. showed a poly(ether‐urethane) solid polymer electrolyte for lithium‐sulfur batteries with excellent interfacial contact [25].

It is worth noting, that most of the works reported so far involve the development of polymer binders for silicon anodes for lithium batteries. Due to the severe volume changes that silicon particles undergo during cycling, self‐healing polymers are particularly attractive for increasing the cyclability of the batteries [26]. As poly(acrylic acid) is a water processable binder that works quite well in the case of silicon anodes, different groups have tried to impart self‐healing properties to it. As illustrative examples, Yang and Song et al. have shown, respectively, that the combinations of acrylic acid‐based polymers with polyurethanes or tannic acid‐based materials respectively resulted in excellent multifunctional polymeric networks, which retain 97% of their initial capacity over 100 cycles when applied as silicon anode binders [27, 28]. Similarly, recent work by Zhou et al. has shown that the combination of poly(acrylic acid) with poly(ether‐thioureas) leads to a universal battery binder featuring soft and hard segments for high‐capacity electrodes [29]. The adoption of self‐healing polymers also addresses the growing demand for durable and safe materials in flexible and wearable energy storage devices, where mechanical deformations are common [30, 31].

Although less common in the scientific literature, recent publications have targeted development of sustainable polymeric binders for cathodes to substitute reference poly(vinylidene fluoride) (PVDF) binders, thereby decreasing fluorine content and substituting the use of toxic *N*‐methylpyrrolidone (NMP) solvent with water during electrode fabrication [33, 34]. However, there is still room for self‐healing polymer binders for cathodes, which are especially necessary to address the sustainability problems that PVDF shows. In this sense, for high‐voltage nickel‐rich cathodes, advanced self‐healing binders could mitigate problems, such as interfacial degradation and transition metal degradation, which prevents electrolyte decomposition [36, 37]. Thus, studies are shifting toward binders that serve as more than just glue, incorporating ionic conductivity to improve rate performance or water‐based processing for sustainable manufacturing [38].

Herein, we report a novel family of self‐healing polymers based on polyampholyte copolymers that synergistically combine ionic interactions, hydrogen bonding, and tunable glass transition temperatures (*T*_g_) to achieve autonomous repair. A series of polyampholyte random copolymers between a dual ionic monomer (DIM) based on methacrylic acid (MA) and dimethylaminoethyl methacrylate (DMAEMA) and poly(ethylene glycol) methyl ether methacrylate (PEGMEMA) are synthetized. The ionic comonomers may impart hardness and rigidity through strong hydrogen bonding and ionic interactions, whereas the plasticizing‐based PEGMEMA segment should lower the *T*_g_, enhancing chain mobility, adhesiveness, and flexibility. The self‐healing ability of the new polyampholyte copolymer family and the influence of the copolymer composition were investigated at different temperatures. Finally, the electrochemical stability and potential application as aqueous binders in cathodes for lithium batteries were investigated to assess their potential for improving electrode durability and cell cycling.

## 2 Experimental Section

### 2.1 Materials

MA (>99%, stabilized with hydroquinone monoethylether), 2‐(dimethylamino)ethyl methacrylate (DMAEMA, >99%, stabilized with hydroquinone monoethylether), PEGMEMA (average M_*n*_ 500), 2,2‐Azobis(2‐methylpropionamidine) dyhydrochloride (AIBA, 97%), PVDF, conductive carbon black (C65) and 1‐methyl‐2‐pyrrolidone (NMP) were purchased from Sigma Aldrich. Lithium iron phosphate (LiFePO4, LFP) was purchased from MSE Supplies. All chemicals were used without further purification.

### 2.2 Synthesis of the Poly(MA‐Co‐DMAEMA‐Co‐PEGMEMA) Copolymers

**FIGURE 1:**
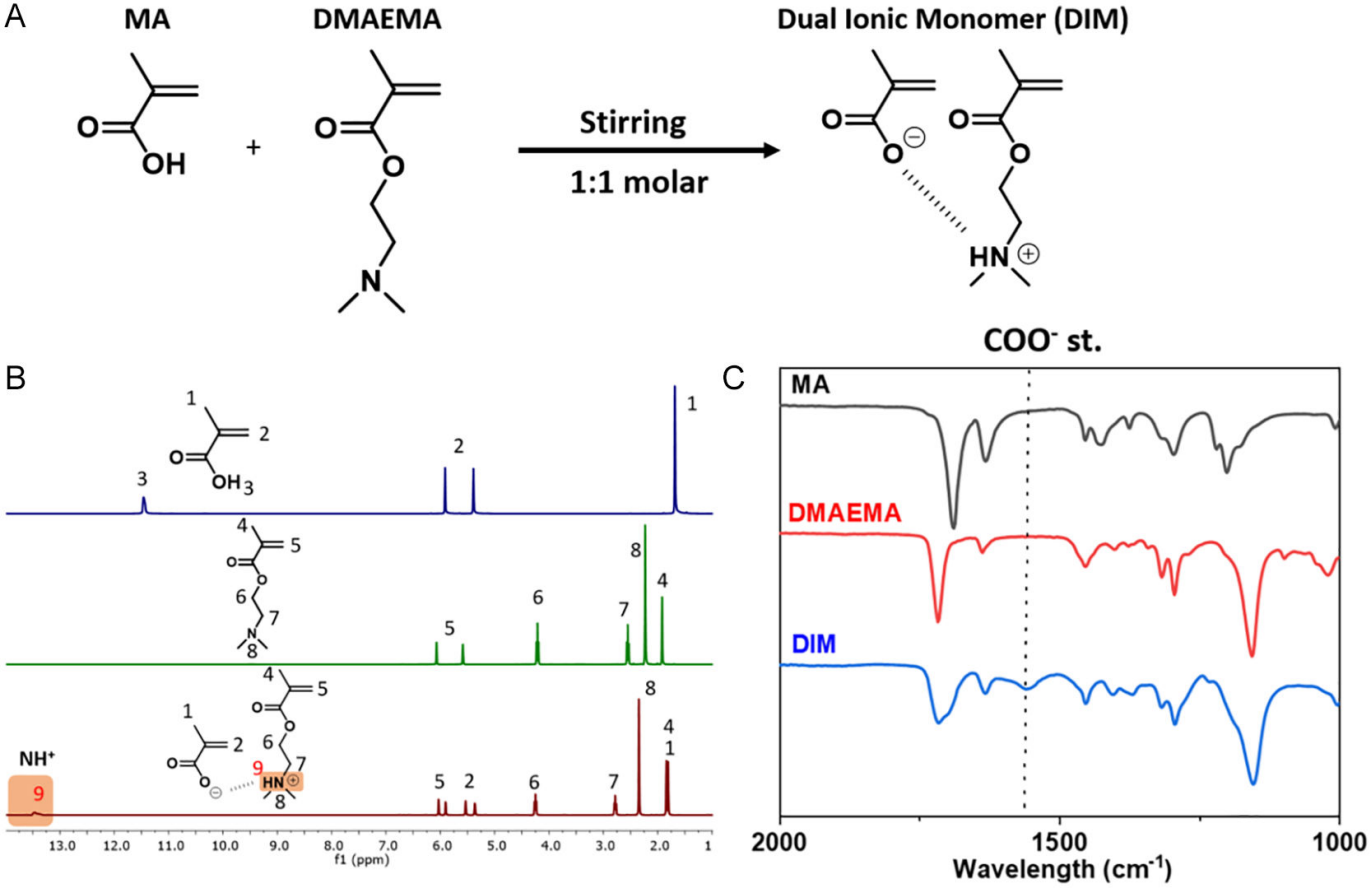
(A) Chemical reaction of the DIM formation. (B) ^1^H‐NMR spectra of MA, DMAEMA, and DIM. (C) FTIR spectra of MA, DMAEMA, and DIM.

Poly(MA‐co‐DMAEMA‐co‐PEGMEMA) copolymers were synthetized via free‐radical polymerization in aqueous media. The first step involves equimolar mixing (1:1 molar ratio) of MA and the 2‐(dimethyl amino)ethyl methacrylate (DMAEMA) by simple stirring for 10 min at room temperature. This is done to ensure correct ionization of the monomers, forming what is called a dual ion monomer (DIM), as illustrated in Figure 1A. In parallel, PEGMEMA is dissolved in distilled water. Various DIM:PEGMEMA molar ratios were chosen to synthetize copolymers including a pure dual ion polymer (poly(DIM)) and a poly(PEGMEMA) homopolymer as reference. The molar ratios indicated in Table 1, correspond to what is fed to the initial system, prior to polymerization.

**TABLE 1:** Copolymer characterization results, including mol ratio, DSC, DMTA, and GPC measurements.

Sample	DIM:PEGMEMA (Mol ratio)	DSC		DMTA		GPC	
		*T*_g, 1_, °C	*T*_g, 2_, °C	*T*_g, 1_, °C	*T*_g, 2_, °C	*M*_w_, KDa	PD
Poly(DIM)	1:0	—	166	—	—	12.0	2.35
C1	0.67:0.33	−45	66	—	—	32.8	3.33
C2	0.58:0.42	−47	55	−41	51	30	3.27
C3	0.47:0.53	−49	48	−44	31	52.5	3.68
C4	0.34:0.66	−51	—	—	13	273.6	10.05
C5	0.19:0.81	−55	—	—	7	482	14.48
Poly(PEGMEMA)	0:1	−57	—	—	—	164.3	6.9

**FIGURE 2:**
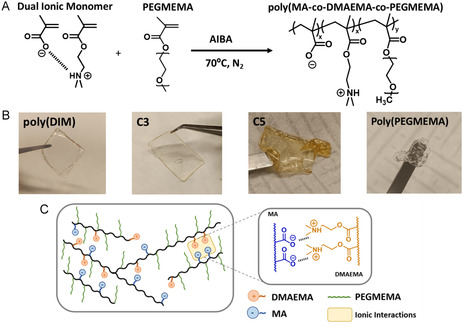
(A) Thermal polymerization scheme. (B) Pictures of poly(DIM), C3, C5, and poly(PEGMEMA) polymeric membranes. (C) Schematic illustration of the synthetized polymeric structures and their chemical interactions.

The total monomer concentration is 5% *w*/*w* in water to prevent excessive increase of viscosity or heat generation during the reaction. 2,2´‐ AIBA is employed as a thermal initiator, added at a concentration of 2.5% (*w*/*w*) relative to the total monomer mass. AIBA is initially dissolved in the PEGMEMA aqueous solution, and this mixture is then added to the previously formed DIM under continuous stirring. The resulting solution is deoxygenated by nitrogen bubbling for 10 min to eliminate dissolved oxygen which could inhibit radical polymerization. To maintain an oxygen‐free environment throughout the reaction, a balloon filled with nitrogen gas is connected to the reaction flask via a needle. Figure 2A presents the polymerization scheme. The solution is placed into a preheated oil bath to initiate the polymerization at 70°C for 7 h. Upon completion, the polymeric solution is transferred into dialysis membranes to remove unreacted species. The dialysis process is carried out for 2–3 days, until complete conversion is confirmed by ^1^H‐NMR Spectroscopy.

### 2.3 Membrane Preparation

Polymeric membranes are prepared by the solvent casting method. First, the previously purified polymeric aqueous solution is poured into a silicone mold and left drying in the fume hood for 2–3 days, allowing for the gradual evaporation of water. Once the membranes are homogeneously formed, they are put into a vacuum oven at 60°C overnight, to ensure complete removal of the remaining solvent. The final appearance of the membranes is depicted in Figure 2B.

### 2.4 Polymer Characterization

Proton nuclear magnetic resonance (^1^H NMR) spectra were recorded in a Bruker Avance DPX 300 at 300.16 MHz. MA, DMAEMA, and the formed DIM were characterized with deuterated DMSO (DMSO‐d_6_) as the solvent, whereas the polymers were analyzed with deuterated water (D_2_O). Polymers were measured just after polymerization, and on each day of the dialysis cleaning process.

Fourier transform infrared (FTIR) spectra were recorded on a Bruker Alpha II spectrophotometer employing a platinum ATR module with a diamond window. All spectra were collected in the range of 4000–500 cm^−1^ with a resolution of 4 cm^−1^ and 64 scans.

Thermal properties of the polymeric membranes were measured by thermogravimetric analysis (TGA) and differential scanning calorimetry (DSC). TGA measurements were carried out on a PerkinElmer Thermogravimetric Analyzer TGA 8000 instrument. Approximately 10 mg of sample was heated from 40°C to 800°C at a heating rate of 10°C/min under a nitrogen atmosphere to evaluate thermal stability.

In contrast, DSC experiments were performed on a Perkin Elmer 8000 DSC equipped with an Intracooler II. Samples of roughly 5 mg were crimped in a nonrecyclable aluminum hermetic pans and subjected to two thermal cycles under nitrogen flow. Each cycled consisted of a cooling step to −90°C and was kept isothermally for 3 minutes, before heating to 150°C, both at a rate of 20°C/min. The second heating scan is plotted to determine the glass transition temperature (*T*_g_) of the samples.

Dynamic mechanical thermal analysis (DMTA) was selected to study viscoelastic properties using an ARES G1 torsion rheometer (TA Instruments) with a parallel plate geometry of 12 mm diameter, at a rate of 3°C/min, in a temperature range from −55°C to 150°C, with a constant frequency of 1 Hz and deformations in the linear region. The glass transition temperature (*T*_g_) has been determined at the maximum of tan *δ*.

Gel permeation chromatography (GPC) was employed to determine the molar mass of the ionic copolymers using a Waters device with three ultrahydrogel columns in series at 35°C (2000, 200, 120 Å). The mobile phase was 0.1 M LiCl, the flow rate was 0.6 mL/min, and the sample concentration was 1 mg/mL. The standards used for the calibration were PEG/PEO (1470–689,500 g/mol).

Adhesion measurements were performed using a TA HD plus Texture Analyzer equipment (Stable Micro Systems), in what is called probe‐tack test. Polymer coatings were prepared by applying them onto a glass support, with an average thickness of 250 µm. In the probe tack, a planar Delrin probe (5 mm diameter) comes into contact with the sample at a test speed of 0.5 mm/s. A 70 N force is applied for a contact time of 1 s before the probe is removed from the polymer film at a controlled velocity. The adhesive stress and strain are obtained, and the adhesive energy is calculated from the area under the stress–strain curve and the sample thickness.

### 2.5 Self‐Healing Characterization

The self‐healing ability of the different copolymers was studied at various temperatures using an OLYMPUS BX51 polarized light microscope fitted with an OLYMPUS SC50 camera and a Mettler FP82HT hot stage with liquid nitrogen cooling capability. A small piece of membrane was cut with a scalpel and placed under the microscope. Before starting the experiment, an initial image was captured. Then, the sample was heated to a specific temperature using a Linkam thermal stage. Micrographs were obtained from the microscope at different times, until the material was fully healed or showed no notable changes.

Rheological measurements were performed with a Discovery Hybrid Rheometer HR10 (TA Instruments) equipped with a parallel plate–plate configuration, and using a stainless steel geometry of 20 mm diameter and 200 µm gap. Oscillatory strain sweeps were carried out from 0.01% to 100% strain at a fixed frequency of 1 Hz and 25°C to determine the linear viscoelastic region (LVR). Frequency tests were conducted from 0.01 to 100 Hz at 1% strain and 25°C to evaluate the frequency‐dependant viscoelastic behavior. The self‐healing behavior was assessed through temperature sweeps by comparing the properties of pristine and intentionally scratched polymer membranes. These tests were carried out at 1 Hz and 1% strain by applying different heating and cooling ramps. For pristine samples, a first heating step from 25°C to 80°C was performed, followed by a cooling step from 80°C to 25°C, at constant heating and cooling rates of 10°C/min. For scratched membranes, the self‐healing behavior was studied by first heating the samples from 25°C to 80°C at 10°C/min . Upon reaching 80°C, samples were maintained at 80°C for 2 h to allow self‐repaire. Subsequently, samples were cooled from 80°C to 25°C at 10°C/min.

### 2.6 Electrode Preparation and Cell Assembly

Cathodes were prepared using LiFePO_4_ (LFP), conductive carbon black (C65) and various binder formulations in a weight ratio of 90:5:5. Five different binders were studied: a reference PVDF dissolved in NMP and four water‐processable binders containing PEGMEMA and/or copolymers (C3, C4, and C5). For each formulation, 3 g of total solids were prepared for the slurry. The polymer binder was first dissolved in its respective solvent (NMP or water) using a final solid‐to‐liquid ratio of 1:0.6, and mixed with a FlackTek speed mixer at 3000 rpm for 5 min. C65 and LFP powders were then sequentially added and mixed thoroughly for 5 min after each addition.

The resulting slurries were cast onto carbon‐coated aluminum (CC‐Al) foil using a doctor‐blade (90 mm/min), dried overnight under vacuum at 60°C, and punched into 8 mm diameter disks. Coin cells (CR2032, Hohsen) were assembled in an argon‐filled glove box using lithium metal (100 μm thick, 10 mm diameter) as the counter/reference electrode, 16 mm glass fiber separators, and 100 μL of 1 M LiPF_6_ in EC:DMC (1:1 *v*/*v*, LP30, Sigma–Aldrich) as electrolyte. The LFP mass loading was approximately 1.5–1.6 mAh/cm^2^.

Galvanostatic cycling was performed using a BTS4000 battery cycler (NEWARE) within a voltage window of 2.8–4.0 V (vs. Li/Li^+^) at room temperature. After a single formation cycle at 0.1 C, rate capability tests were conducted by charging and discharging at 0.5, 1, 2, 3, and 5 C for 5 cycles each. Cells were subsequently returned to 1 C for an additional 100 cycles. Each test was repeated with at least three cells, and the capacity data showed a standard deviation below 5%.

### 2.7 Electrochemical Characterization

Electrochemical impedance spectroscopy (EIS) was employed to determine the ionic conductivity of the ionic copolymer membranes using an Autolab 302N potentiostat at different temperatures from 100°C to 20°C at every 10°C intervals. The measurements were obtained in the range between 300 kHz to 1 Hz, with a perturbation amplitude of 20 mV. A symmetrical stainless steel/polymer membrane/stainless steel cell was assembled. The surface area was 0.5 cm^2^, and the thickness was measured after each measurement.

The Ohmic resistance of the polymer membranes, obtained from the Nyquist plot at the low frequency end of the semicircle, was used to calculate the ionic conductivity using the following equation, where *σ* is the value of ionic conductivity, *R* is the material resistance, *l* is the thickness of the membrane, and *A* is the area of the electrode.

$$\text{σ}=\frac{1}{R}\times \frac{l}{A}$$

To evaluate the electrochemical stability of the binders, cyclic voltammetry (CV) measurements were performed on composite electrodes composed of binder and C65 in a 1:1 weight ratio (50:50 wt.%). The slurries were prepared following the same procedure as for the LFP cathodes, coated on CC‐Al foil, and punched into 8 mm disks. These binder‐only electrodes were assembled into coin cells with lithium foil as the counter/reference electrode and 100 μL of LP30 electrolyte. After 8 h of stabilization at open‐circuit potential, CVs were conducted on a VMP‐3 potentiostat (Bio‐Logic Science Instruments) at a scan rate of 0.1 mV/s. The voltage range was increased sequentially for each cell from 2.8 to 4.0 to 2.8–4.1, 2.8–4.2, 2.8–4.3, and up to 2.8–4.5 V (vs. Li/Li^+^), to progressively evaluate the oxidative stability and detect any redox activity or irreversible processes in the binders.

## 3 Results and Discussion

Prior to copolymerization, a DIM is synthesized by proton transfer neutralization of MA and DMAEMA. Thus, MA and DMAEMA monomers are mixed in a 1:1 molar ratio, exhibiting a slight increase in viscosity indicating an exothermic acid–base reaction. In this step, a proton (H^+^) is transferred from the carboxylic acid moiety of the MA, to the amine group of DMAEMA, forming an ionic interaction referred to as the DIM with a carboxylate anion (COO^−^) and a protonated tertiary amine cation (NH^+^), as shown in Figure 1A.

The formation of DIM is confirmed by ^1^H‐NMR and FTIR spectroscopy. In Figure 1B, not only the ^1^H NMR of both pure monomers but also that of the formed dual ionic structure are presented. Regarding the pure monomers, MA displays a ─CH_3_ signal at 1.67 ppm, both acrylic peaks at 5.39 and 5.91 ppm, and the ─OH moiety at 11.46 ppm. In the case of DMAEMA, the methacrylate ─CH_3_ is at 1.92 ppm, while both ─CH_3_ groups linked to the amine resonate at 2.23 ppm. CH_2_ groups linked to the nitrogen and oxygen appear at 2.55 and 4.21 ppm, respectively, and both acrylic peaks at 5.59 and 6.07 ppm. When both monomers are mixed, chemical shifts are observed due to the proton transfer. The methacrylate methyl group of MA is shifted downfield, appearing at a higher chemical shift of 1.82 ppm, whereas the DMAEMA methyl peak is slightly shifted upfield, to a lower chemical shift of 1.84 ppm. The two ─CH_3_ groups bonded to the amine group in DMAEMA are slightly shifted to 2.34 ppm, while ─CH_2_ groups covalently linked to the amine and oxygen appear at 2.78 and 4.25 ppm, respectively, what means that they are downfield moved to higher chemical shifts. The four acrylic protons of both MA and DMAEMA resonate in the range 5.36–6.03 ppm, similar that for neutral monomers. Notably, the formed NH^+^ cation appears at 13.45 ppm, while the ─OH peak disappears compared to the MA spectrum, what confirms proton transfer to the amine group and consequently the formation of the targeted ionic structure, by electrostatic and hydrogen bonding interactions. Every peak was consistent with the expected stoichiometry.

The zoomed FTIR spectra in the wavelength range of 2000–1000 cm^−1^ is presented in Figure 1C, for MA, DMAEMA, and the resulting DIM, whereas the complete spectra can be found in Supporting Information (SI) Figure S1. Related to pure monomers, a new absorption band appears at 1558 cm^−1^ in the spectrum of DIM, which belongs to the asymmetric stretching of the carboxylate (─COO^−^), which also confirms by FTIR that the acid–base reaction took place, leading to the formation of DIM [39].

Then, a series of random copolymers were synthesized via thermal aqueous free‐radical polymerization of the aforementioned DIM and PEGMEMA monomer at different monomer ratios (Table 1), as shown in Figure 2A. After the polymerization reaction, the copolymers were purified by a dialysis process. Complete monomer purification was confirmed by ^1^H NMR spectroscopy (Figure S2), in which no signals corresponding to acrylic moieties are observed in the range of 5–6.55 ppm indicating successful removal of unreacted monomers. FTIR spectra of the DIM and polyampholyte homopolymer poly(DIM) are plotted in Figure S3, where the disappearance of the C&dbond;C stretching band (1630 cm^−1^) and &dbond;C─H stretching band (930 cm^−1^) confirms the successful polymerization. Apart from the peaks confirming the polymerization, FTIR signals associated with both carboxylate anion (1550 cm^−1^) as well as the carbonyl stretching for both MA and DMAEMA structures (1750 cm^−1^) are identified.

Copolymer membranes were then obtained via solvent casting in water. Depending on the composition the aspect of the membranes ranged from rigid and brittle (high DIM content), to soft and elastic (high PEGMEMA content). The corresponding copolymers are named from C1 to C5, ranging in molar ratios of DIM:PEGMEMA from 0.67:0.33 for C1 (highest DIM ratio) to 0.19:0.81 for C5 (highest PEGMEMA ratio). Figure 2B, shows that poly(DIM) yields a brittle and self‐standing membrane, while increasing PEGMEMA content leads to progressively softer and more deformable membranes, as C3 and C5 copolymer compositions illustrate. In contrast, PEGMEMA homopolymer forms a highly adhesive membrane with poor self‐standing capacity. The molecular weight was determined by GPC (Tables 1 and S1), showing an increase with the PEGMEMA content, varying from 12.000 gr/mol for poly(DIM) homopolymer to 482.000 gr/mol for C5 copolymer. It should also be noted that high dispersity values obtained by free radical polymerization limits the level of detailed quantitative structure‐property correlations that can be taken. However, as all copolymers follow identical synthesis and characterization conditions with similar molecular weight ranges, comparative trends across the copolymer series are reliable for properties such as ionic conductivity or self‐healing efficiency.

A schematic illustration of the prepared materials is depicted in Figure 2C, highlighting the supramolecular interactions between carboxylate and amine chemical motifs. Both hydrogen bonding and electrostatic interactions may occur between positively charged polymerized ammonium pendants and negatively charged carboxylate groups from the acrylate moiety. Those supramolecular interactions contribute to the membrane hardening, with higher ionic content leading to brittle membranes due to the strong ionic crosslinking. In contrast, higher PEGMEMA content reduces the self‐stability of the material, but enhances key properties such as adhesion or deformability.

**FIGURE 3:**
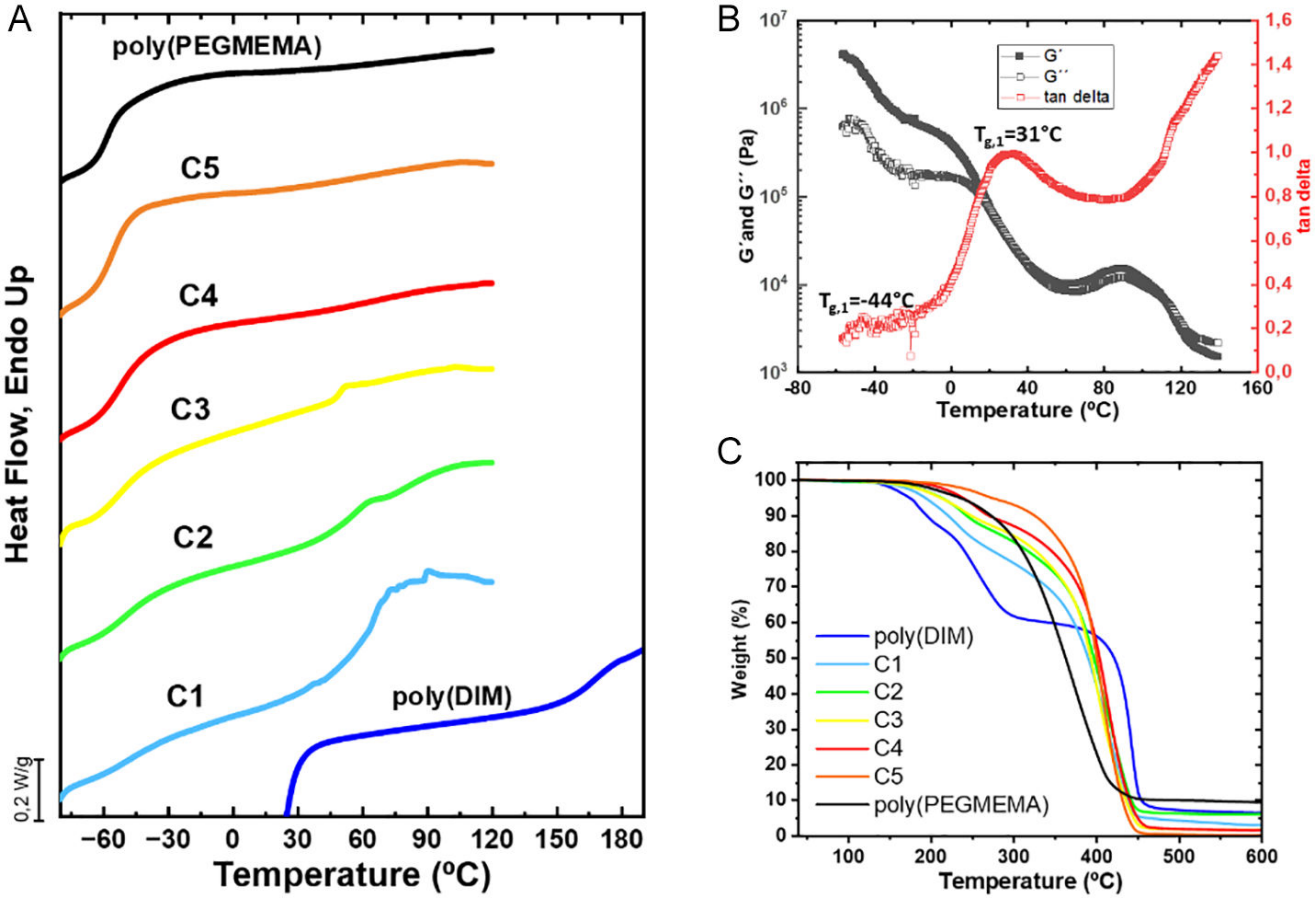
Thermal characterization of the copolymers. (A) Differential scanning calorimetry (DSC) spectra. (B) Dynamic mechanical analysis (DMTA) for C3 copolymers. (C) Thermogravimetric analysis (TGA).

DSC determined glass transition temperatures of the copolymers as shown in Figure 3A. Both pure poly(DIM) and poly(PEGMEMA) display single glass transition temperature behaviors, low *T*_g_ of −57°C for the elastic poly(PEGMEMA) and high *T*_g_ of 166°C for the brittle poly(DIM), consistent with their mechanical properties. In contrast, random copolymers exhibit two distinct glass transitions, indicative of a biphasic system. The lower *T*_g_ is attributed to PEGMEMA‐rich phase, while the higher *T*_g_ phase is attributed to ionic phase, what suggest partial phase separation between components. All *T*_g_ values derived from DSC measurements are summarized in Table 1.

Focusing on low *T*_g, 1_, DSC analysis shows a progressive increase from −57°C from poly(PEGMEMA) to −45°C for C1 material. This trend reflects gradual rigidity increase as the ratio of DIM composition increases in the copolymer, resulting in less stretchable and harder membranes. In contrast, related to higher *T*_g, 2_, the opposite behavior is observed: the glass transition temperature decreases as DIM:PEGMEMA ratio decreases in the system. This higher *T*_g_ value drops from 166°C for the poly(DIM) homopolymer, to 48°C for C3 homopolymer, revealing the softening of the materials associated with the internal plasticization of the PEGMEMA comonomer.

It should be noted that the second glass transition temperature, associated with the ionic component, could not be clearly detected by DSC in samples C4 and C5. Therefore, DMTA was performed to determine this second *T*_g_ of those membranes. The obtained results for DMTA are listed in Table 1 and plotted in Figure S4. DMTA analysis corroborates these trends, although the absolute value of the glass transition temperature slightly differs, due to the differences between DMTA and DSC methodologies. By DMTA, low *T*_g_ is not detected for softest membranes, values increase from −44°C for C3 to −41°C for C1. However, high *T*_g_ values decrease from 51°C for C2 membranes to 7°C for C5 membranes. The DMTA results for C3 copolymer are shown in Figure 3B.

The thermal stability of the polymer membranes was studied by TGA in order to determine the operational temperature range and the stability profiles, which are plotted in Figure 3C. Decomposition temperature values of 5% weight loss (*T*_d5%_) and maximum decomposition temperatures (*T*_dmax_) are listed in Table S2, and range from 174°C to 273°C and from 253°C to 441°C, respectively. The lowest *T*_d5%_ corresponds to pure poly(DIM) and increases with higher PEGMEMA content in the system, which means that these materials start degradation at higher temperatures. Regarding degradation steps, poly(PEGMEMA) displays a unique degradation step with a maximum *T*_dmax_ at 370°C, whereas poly(DIM) shows a two‐step degradation, probably linked to both monomers in its structure, with two *T*_dmax_ values of 253°C and 441°C for both degradation decays. In contrast, copolymers reveal a combined degradation trend, with two weight drops, the first one attributed to the ionic part and the second one to PEGMEMA, whereas this second decay is much more pronounced. However, copolymers show a unique *T*_dmax_ value ranging from 415°C to 405°C.

Self‐healing capacity of the materials was evaluated through different techniques, including rheological temperature sweeps and optical microscopy tests. For this purpose, polymeric membranes were scratched before monitoring them by the optical microscope at diverse temperatures and healing times. As mentioned, there are two key parameters considered, the self‐healing temperature, defined as the minimum temperature at which the polymer undergoes healing, and the self‐healing time, defined as the minimum time the material fully heals. Notably, poly(DIM) homopolymer membrane was too brittle even to try self‐healing experiment, and poly(PEGMEMA) lacked mechanical stability to form a homogeneous polymer membrane. Self‐healing results of the different copolymers are reported in the Table 2.

**TABLE 2:** Summary of the self‐healing capacity of copolymer membranes at different times and temperatures.

Sample	Temperature, °C	Self‐ healing time	Self‐healing?
C1	80 (*T*_g_ + 14)	24 h	No
C2	80 (*T*_g_ + 25)	21 h	Partially
C3	80 (*T*_g_ + 32)	2 h	Yes
	50 (*T*_g_ + 2)	12 h	Yes
	40 (*T*_g_ – 8)	24 h	Partially
C4	70	15 min	Yes
	50	6 h	Yes
	40	12 h	Yes
C5	50	4 h	Yes
	40	12 h	Partially

**FIGURE 4:**
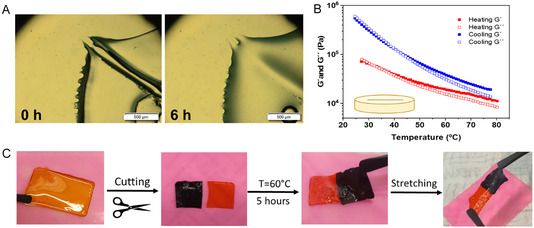
Self‐healing capacity of the copolymers. (A) Optical microscope illustration of C4 copolymer. (B) Rheological temperature sweeps for the scratched C3 membrane. (C) Visual illustration of the self‐healing process in C4 membranes.

In relation to optical microscopy results, the images taken for the different copolymers are shown in Figures S5–S9, where the healing temperature was decreased as well as the *T*_g_ values of C1–C5 polymers. As observed, C1 copolymer exhibits no self‐healing capacity even at 80°C and 24 h, the maximum time and temperatures considered. There are no visible modifications after that time, indicating a lack of polymer chain mobility and dynamic interactions. However, a partial self‐healing is observed for the C2 membrane. Although there is not complete closure, partial healing indicates limited rearrangement and segmental motion at high temperatures. The healable behavior of C3 membrane is also studied, taking into account that the *T*_g_ is 48°C. The first measuring temperature was 80°C, but the made scratch fully healed after only 2 h. If the temperature is dropped to 50°C the material also recovers from the made damage but in 12 h. Finally, temperature was decreased to 40°C, but in this case, only a partial healing occurs, probably because the *T*_g_ of the material is above the healing temperature. Following with C4, a fast healing of only 15 min is obtained at 70°C, whereas at healing temperatures of 50°C and 40°C the self‐healing times were 6 and 12 h respectively. Self‐healing capacity for C4 membrane at 50°C is shown in Figure 4A. Finally, C5 based copolymer was able to completely heal in 4 and 12 h at self‐healing temperatures of 50°C and 40°C respectively. It must be mentioned that images taken for C4 and C5 copolymer exhibit limited resolution, because the materials lack consistency and are highly stretchable and adhesive, which makes it difficult to make a clean scratch.

Rheological tests were carried out on both as prepared and intentionally scratched C3 (53%mol PEGMEMA) and C4 (66%mol PEGMEMA) membranes. For the as prepared C3 copolymer membrane, the amplitude sweep (Figure S10A) revealed a predominantly solid‐like behavior at room temperature, with the storage modulus (*G*´ ≈ 6·10^4^ Pa) exceeding the loss modulus (*G*’’ ≈ 4·10^4^ Pa) within the LVR up to 30% strain. Frequency sweeps conducted within the LVR (Figure S10B) showed a transition toward fluid‐like behavior, as the gap between *G*’ and *G*’’ decreased with increasing frequency. This indicates that the material shifted from a solid‐like to a more liquid‐like state, likely because higher frequencies limited the time available for molecular relaxation, resulting in an apparent increase in both moduli. Temperature sweeps (Figure S10C) demonstrated a decrease in both moduli with rising temperature, reflecting enhanced polymer chain mobility. At 80°C, the widening gap between *G*’ and *G*’’ suggested a more pronounced viscoelastic character and increased flexibility. Upon cooling, this trend was reversed, *G*´ increased, indicating that the polymer chains gained rigidity, as both moduli rose to ≈10^5^ Pa, accompanied by a reduced gap between them. This behavior may suggest partial rearrangement of the polymer chains, producing a more brittle and less crosslinked membrane structure. The same thermal protocol was applied to the defect‐induced C3 membrane. As shown in Figure 4B, its behavior was slightly different. At low temperatures, the membrane exhibited a more liquid ‐like response (*G*´´ > *G*´), consistent with partial disruption of the crosslinking network. Upon heating, the response of the polymer resembled that of the undamaged membrane, both moduli decreased with temperature, and the gap between them widened, suggesting enhanced chain mobility and reorganization that facilitated network reformation. After maintaining the sample at 80°C for 2 h, both moduli increased, and the scratched membrane recovered the initial *G*’ values characteristic of the undamaged membrane, thus confirming its self‐healing capability. At the end of the cooling cycle, the membrane exhibited even higher moduli (≈10^6^ Pa) than at the beginning of the experiment, similar to the native membranes. A comparable trend was observed for copolymers with a higher PEGMEMA content (sample C4, Figure S11). Increasing PEGMEMA content enhanced membrane flexibility, as evidenced by a slight decrease in *G*’ (≈2·10^4^ Pa) and an extended LVR up to 60% strain (Figure S11A). The frequency dependence of *G*’ and *G*’’ (Figure S11B) closely resembled that of sample C3. Temperature sweeps (Figure S11C) again showed a decrease in both moduli with increasing temperature due to increased polymer mobility. Nevertheless, upon cooling, the trend did not vary, and the sample presented similar *G*’ and *G*’’ values to those observed prior to heating, likely reflecting the greater flexibility and faster chain rearrangement afforded by the higher PEGMEMA content. For the scratched C4 membranes (Figure S11D), the polymer behaved like the native undamaged membranes, both moduli decreased with temperature and recovered upon cooling, ultimately regaining the initial *G*’ value of the undamaged membranes (≈2·10^4^ Pa), thus confirming the self‐healing of the membranes.

The self‐healing process of polymer membranes was also studied in a laboratory visual experiment (Figure 4C). C4 based membranes were previously colored by adding orange and blue inks before the casting method. Membranes were then cut in two half pieces and mixed crossways at 60°C for 5 h. The formed double colored membranes behave like a single polymer, after self‐healing treatment, showing self‐stability and deformability in laboratory stretching tests. Heating the membranes increases chain mobility, enabling the rearrangement of the polymer chains and the reformation of new supramolecular interactions, electrostatic and hydrogen bonding.

**FIGURE 5:**
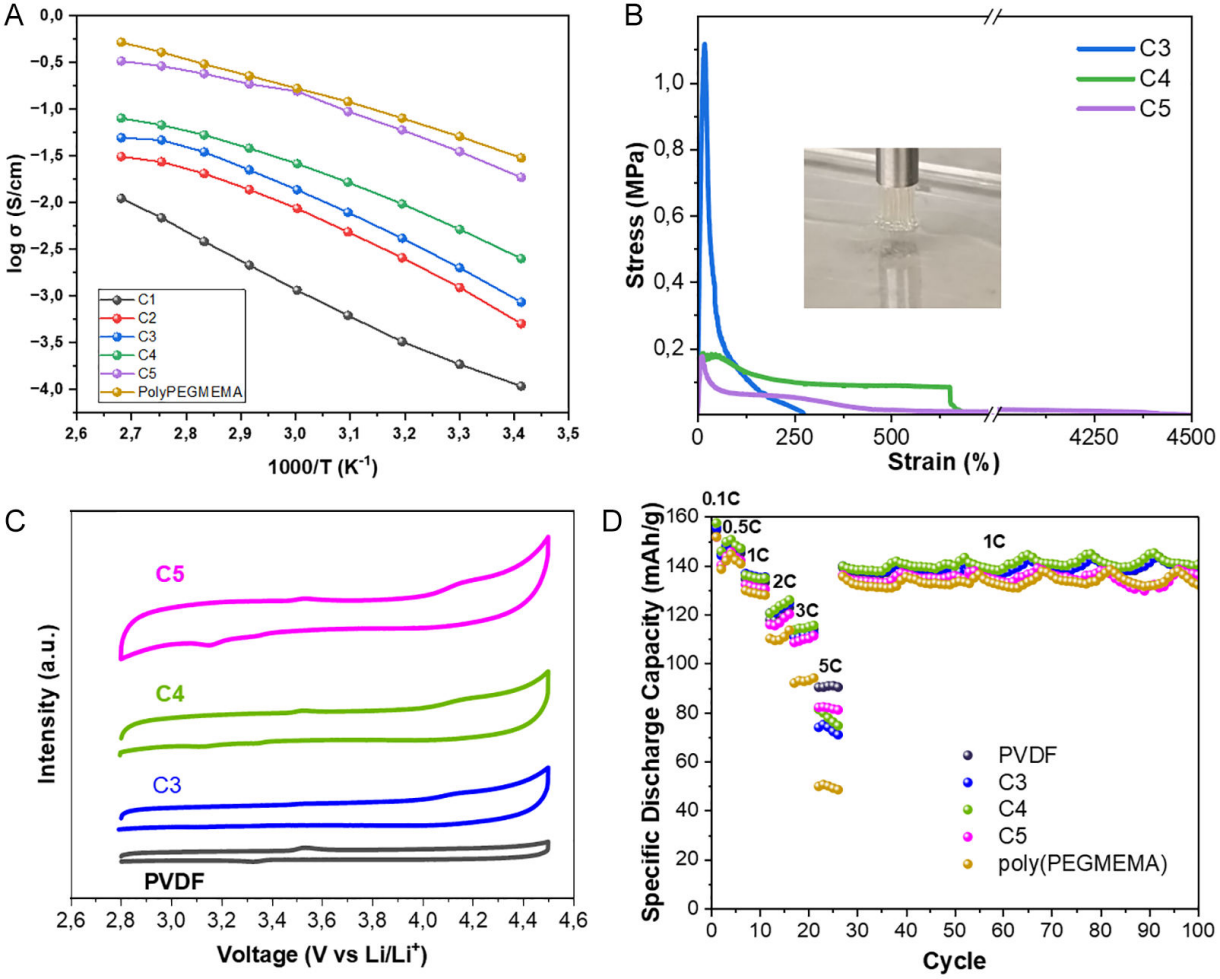
(A) Ionic conductivity measurements of the copolymers. (B) Adhesive probe tack tests for C3, C4, and C5 copolymers. (C) Cyclic voltammetry for the potential electrochemical window of copolymers. (D) Electrochemical performance of LFP coin cells using copolymers as binders at voltage profiles of 0.5, 1, 2, 3, and 5 C.

Focusing on the electrochemical characterization of these materials for their application in batteries, ionic conductivity was measured at the dry state, as it is shown in Figure 5A. Results indicate that the copolymers range from 10^−6^ to 10^−9^ S/cm at room temperature, and the ionic conductivity increases exponentially with the temperature. Making a comparison between copolymers, the ionic conductivity increases with the PEGMEMA content. There are two main parameters affecting ionic conductivity values, the higher ionic content in the system and the low glass transition temperature (*T*_g_). In this case, the higher the PEGMEMA content in the membranes, the less ionic content in the system, but also lower *T*_g_ and higher flexibility and easier ion movement, which results in higher ionic conductivity. During the heating process, the ionic conductivity tendency is constant: C5 > C4 > C3 > C2 > C1, what demonstrates that the lower *T*_g_ is the main factor increasing conductivity in the copolymers. Table S3 summarizes the activation energy values calculated, which range from 53.83 kJ/mol for C1 to 31.95 kJ/mol for poly(PEGMEMA). Lower activation energy means higher ionic conductivity due to the lower energy barrier. In same termss, the more rigid the copolymer membranes are the higher activation energy is required.

Figure 5B displays the adhesive performance of C3, C4 and C5 polymeric membranes, represented by stress–strain plot. C3 membrane exhibits the highest maximum adhesive strength, with a maximum peak of 1.30 MPa, which indicates a strong interfacial bonding. However, the adhesive stress rapidly losses and the membrane fractures at a low strain (274%), revealing a limited deformability and high adhesive stiffness. In contrast, C4 and C5 membranes presented a lower initial maximum adhesive strength (0.27 and 0.24 MPa respectively) but a significantly higher elongation at break, reaching strain values of 718% and 4480% respectively. This pronounced ductility is related to the fibrillation process, where the adhesive layer forms micro fibrils during deformation, enabling load transfer and plastic deformation before detachment, as illustrated in Figure 5B. Turning to the energy of adhesion, it quantifies the ability of a material to resist removal and absorb mechanical energy during the process. According to calculated values (Table S4), C4 is the most resistant and toughest adhesive material, providing higher resistance against mechanical loads, thanks to fibrillation capacity, showing an adhesive energy of 272 J/m^2^. Although different strain deformations, C3 and C5 display similar adhesive energies (127 and 117 J/m^2^), significantly lower compared to C4, indicating that C3 is a strong but brittle material and in contrast, C5 achieves high deformation but low stress levels. Overall, this superior energy dissipation and toughness, makes C4 promising candidate suitable for application in flexible devices as battery components, where sustained mechanical resistance to impact are critical for long‐term performance.

To evaluate the electrochemical stability and activity of the binders, CV measurements were carried out. The full CV for PVDF, C3, C4, and C5 is shown in Figure S12, while Figure 5C overlays the final CV cycle for each binder in the voltage range of 2.8–4.5 V versus Li/Li^+^ to facilitate a direct comparison of their electrochemical behavior. As shown, the CV profile of PVDF exhibits minimal current response over multiple cycles, indicating negligible electrochemical activity, consistent with its known inertness and stability within this voltage window. Similarly, the alternative binders C3, C4, and C5 display comparable low‐current profiles, with no apparent redox peaks or significant current increase over cycling, suggesting that these materials are electrochemically stable and do not undergo parasitic reactions within the examined potential range. Notably, a subtle yet progressive increase in hysteresis with higher PEGMEMA content in the binder composition (C3 < C4 < C5) is observed. This suggests that the incorporation of increasing amounts of PEGMEMA may affect the interfacial properties or ion transport dynamics during polarization, leading to broader loops in the CV profiles. However, these changes remain moderate and do not compromise the overall electrochemical stability, supporting the viability of C3, C4, and C5 as alternative binder systems for lithium‐ion batteries.

The electrochemical performance of these novel ionic polymers as binders was evaluated using LFP|Li half‐cells, with a particular focus on the rate capability and long‐term cycling stability. Rate performance testing was conducted at various C‐rates to assess the binders’ ability to support fast lithium‐ion transport (Figure 5D). At a high discharge rate of 5 C, the PVDF‐based electrode maintains the highest capacity (~90 mAh/g), reflecting its well‐established performance. Among the alternative systems, the C5 binder delivers over 80 mAh/g at 5 C, indicating strong rate capability and efficient ionic transport. C4 and C3 follow closely, demonstrating comparable but slightly reduced performance. In contrast, the poly(PEGMEMA) exhibits a pronounced capacity drop at elevated C‐rates, highlighting the crucial role of the DIM component in enhancing ionic conductivity. As a DIM facilitates improved lithium‐ion mobility and helps minimize polarization under fast charge/discharge conditions. The voltage profiles presented in Figure S13, which illustrate the increased polarization and voltage hysteresis in the poly(PEGMEMA) compared to DIM‐containing binders, further supports this behavior.

Following the rate tests, long‐term cycling was conducted at a constant 1 C rate and room temperature. As shown in Figure 5D, the C3 and C4 formulations exhibit stable cycling behavior and maintain specific discharge capacities comparable to the PVDF reference (~140 mAh/g). These binders retain over 90% of their initial capacity after more than 100 cycles, demonstrating robust mechanical and electrochemical stability. C5 shows good initial performance but begins to degrade beyond 80 cycles, while the poly(PEGMEMA) suffers early and significant capacity fade. This rapid decline is attributed to the lack of both mechanical reinforcement and ionic conduction pathways, reinforcing the critical role of DIM in achieving balanced performance.

Figure 5D reveals an important mismatch between self‐healing functionality and electrochemical performance. C5 exhibits the highest self‐healing efficiency among the synthetized binders, but it shows inferior rate capability compared to C4 and C3. This observation indicates that self‐healing alone does not dominate electrochemical performance; it is a combination of binder properties that governs this behavior. C3 and C4 benefit from stronger adhesion to current collector and active material particles, as evidenced by the higher adhesive stress values in stress–strain curves from Figure 5B. PVDF, although lacking self‐healing properties, maintains competitive rate performance due to its excellent adhesion properties and electrochemical stability. These results suggest that adhesion strength and mechanical integrity are the primary factors governing electrochemical performance, whereas self‐healing is expected to play a more prominent role at long‐term cycle life, particularly under conditions such as mechanical stress or repeated volume expansion. Optimal binder design requires a balance between adhesion, mechanical robustness and self‐healing capability, depending on application requirements.

## 4 Conclusion

In this article, we show the synthesis and characterization of polyampholyte copolymers based on a simple DIM based on the neutralization reaction between MA and dimethyl aminoethyl methacrylate and PEGMEMA as internal plasticizer. The synthesized copolymers range from rigid self‐supporting membranes at high dual ionic comonomer content to elastic and deformable materials at higher PEGMEMA content. Thermal analysis revealed stability until 200°C and varying two glass transition temperatures one at higher temperatures associated with the ionic domains and one below ambient temperature associated to the poly(ethylene oxide) domains. Remarkably, polyampholyte copolymer membranes demonstrate composition dependent self‐healing properties even at temperatures as low as 40°C. Adhesive strengths over 1 MPa and ionic conductivities of 10^−6^ S/cm further support their potential application as water processable cathode binders. Finally, electrochemical characterization demonstrates that lithium iron phosphate cathodes processed using the copolymers as binders present high rate capability and cyclic performance, establishing them as a compelling sustainable alternative to PVDF for advanced battery formulations.

## Supporting Information

Additional supporting information can be found online in the Supporting Information section.

## Funding

This study was supported by HORIZON EUROPE Climate, Energy and Mobility (101104028).

## Conflicts of Interest

The authors declare no conflicts of interest.

## Supporting information

Supplementary Material

## Data Availability

The data that support the findings of this study are available on request from the corresponding author. The data are not publicly available due to privacy or ethical restrictions.
