# Supplementary material for: Polyampholyte Copolymers Based on Dual Ionic Monomers as Self‐Healing Aqueous Binders for Lithium Ion Battery Cathodes
Source: ChemSusChem. 2026 May 12;19(9):e202502470. doi: 10.1002/cssc.202502470 (PMC13168370; doi:10.1002/cssc.202502470)
Supplement: Supplementary file 1 — Supplementary Material [file CSSC-19-e202502470-s001.pdf]

SUPPORTING INFORMATION

Polyampholyte Copolymers based on Dual Ionic Monomers as Self-Healing Aqueous Binders for Lithium Ion Battery Cathodes

Jon López de Lacalle[1], Ana Clara Rolandi[1], Jorge L. Olmedo-Martínez[1], Miryam Criado-Gonzalez[1], Eduardo J. Garcia-Suarez[2], Nerea Casado[1*], David Mecerreyes[1*]

[1] J. Lopez de Lacalle, A.C. Rolandi, J.L Olmedo-Martinez, M. Criado-Gonzalez, N.Casado, D. Mecerreyes
POLYMAT
University of the Basque Country
Avenida Tolosa 72, Donostia-San Sebastián, Spain
nerea.casado@ehu.eus, david.mecerreyes@ehu.eus

[2] E.J. Garcia-Suarez
Center for Cooperative Research on Alternative Energies (CIC energiGUNE)
Basque Research and Technology Alliance (BRTA)
01510, Vitoria-Gasteiz, Spain

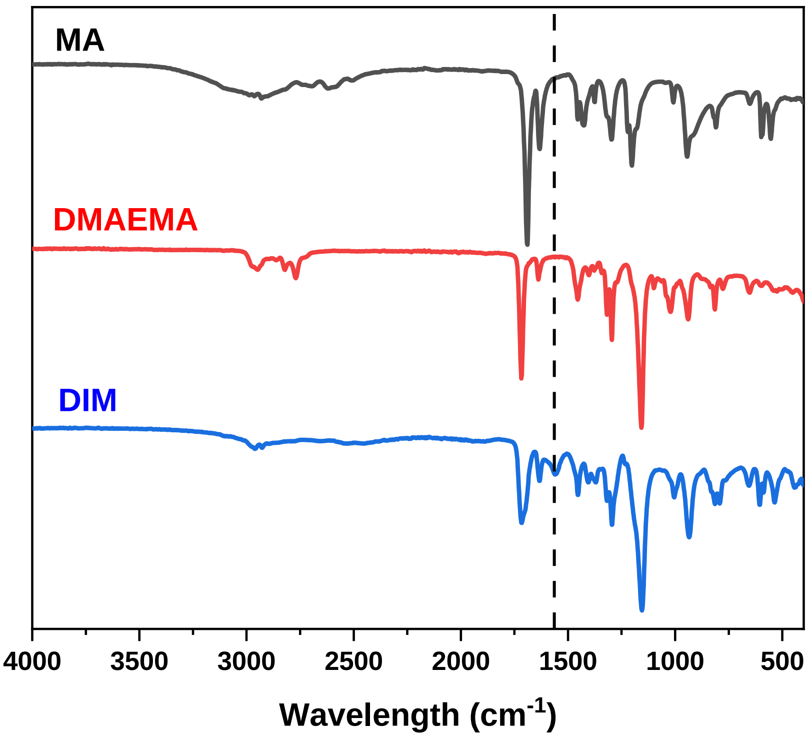

Figure S1. *Complete FTIR spectra for both pure monomers MA and DMAEMA and the formed DIM.*

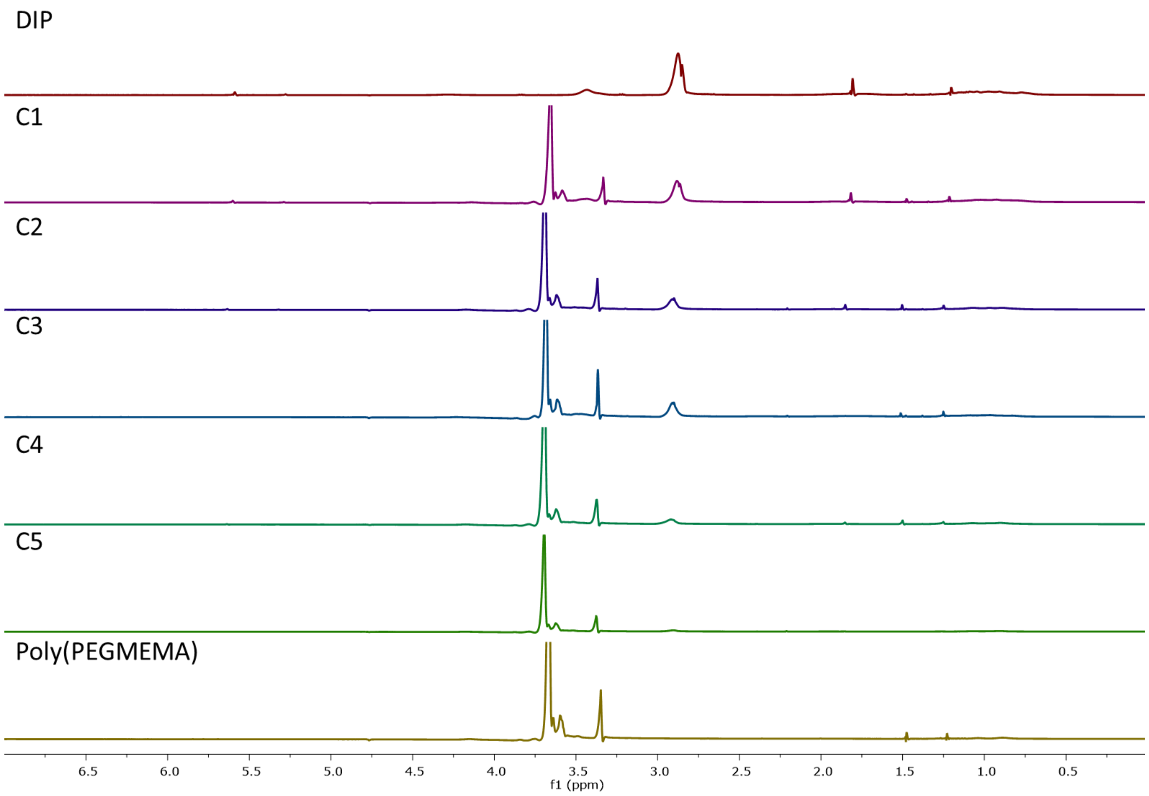

Figure S2. *^{1}H-NMR Spectra for all the synthetized polymers.*

Table S1. *Molar masses of the copolymers obtained by GPC.*

Polymer	Mn (kDa)	Mw (kDa)	PD (Mw/Mn)
polyDIM	5.1	12.0	2.3
C1	9.8	32.8	3.3
C2	9.2	30	3.2
C3	14.3	52.5	3.7
C4	27.2	273.6	10.0
C5	33.3	482.0	14.5
Poly(PEGMEMA)	23.7	164.3	6.93

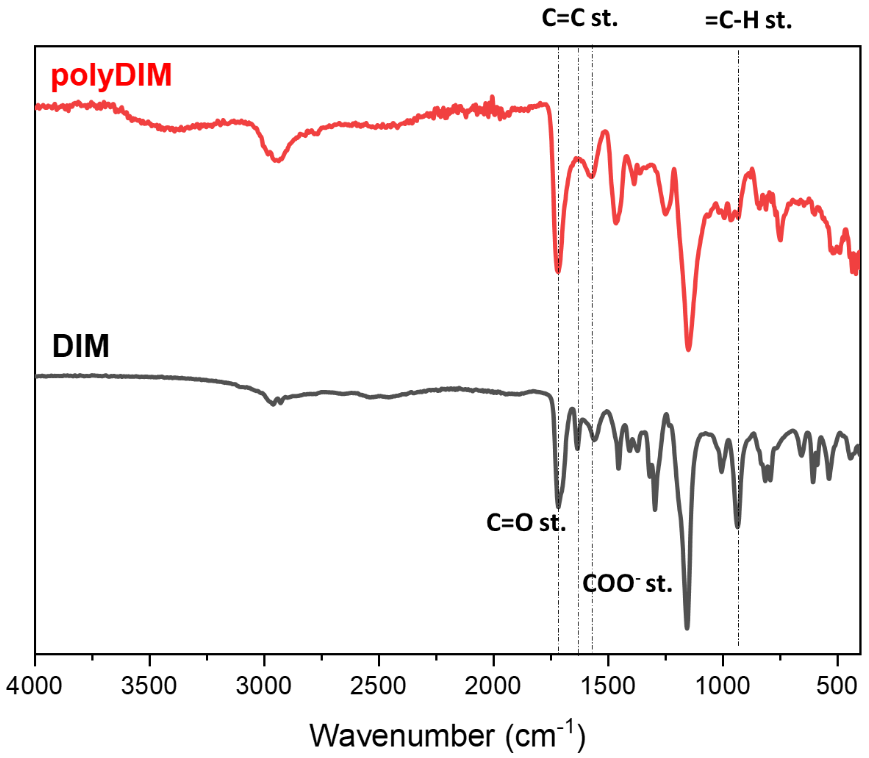

Figure S3. *Comparative of FTIR spectra of dual ionic monomer (DIM) vs dual ionic polymer poly(DIM).*

Table S2. *Thermogravimetric analysis (TGA) of the copolymers. Decomposition temperature values of 5% weight loss ($T_{d5\%}$) and maximum decomposition temperatures (T_{dmax}).*

**Sample**	**$T_{d5\%}$ (°C)**	**T_{dmax} (°C)**	
**DIP**	174.08	253	441
**C1**	192.85	415	
**C2**	212.63	413	
**C3**	211.44	400	
**C4**	238.66	413	
**C5**	273.28	405	
**Poly(PEGMEMA)**	235.03	370	

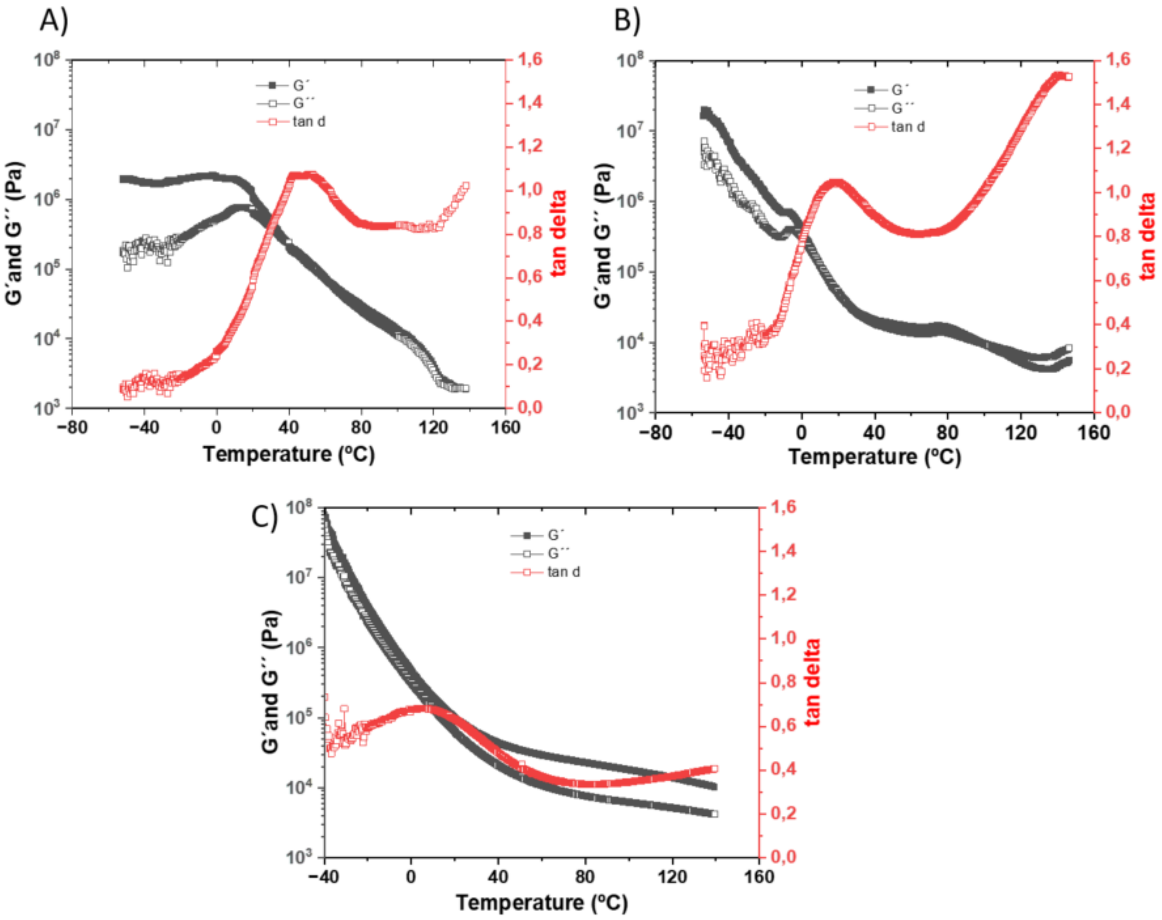

Figure S4. *Dynamic Mechanical Thermal Analysis (DMTA) for synthetized copolymers A) C2 B) C4 and C) C5.*

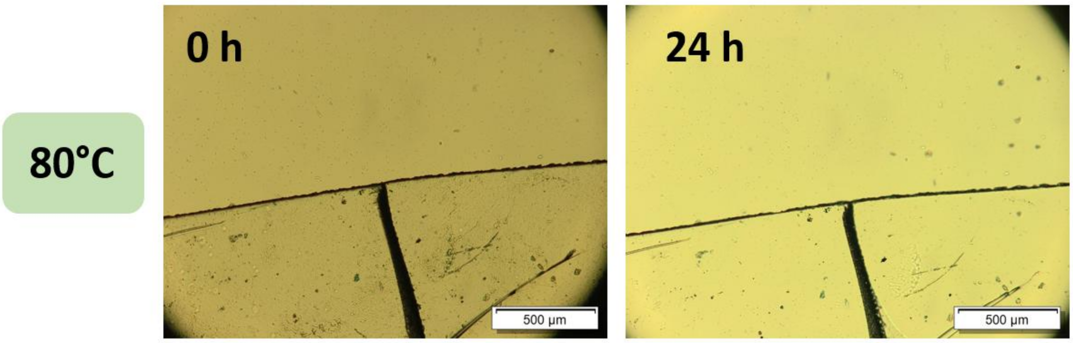

Figure S5. *Self-healing measurements for the C1 polymer membranes at time 0 and after 24 hours at 80°C.*

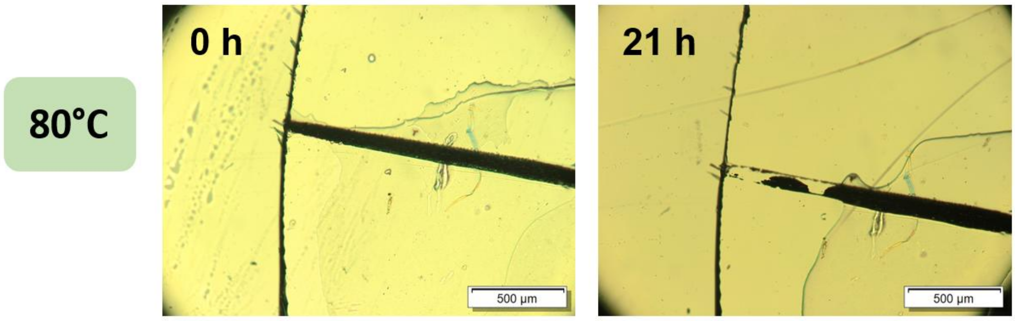

Figure S6. *Self-healing measurements for the C2 polymer membranes at time 0 and after 21 hours at 80°C.*

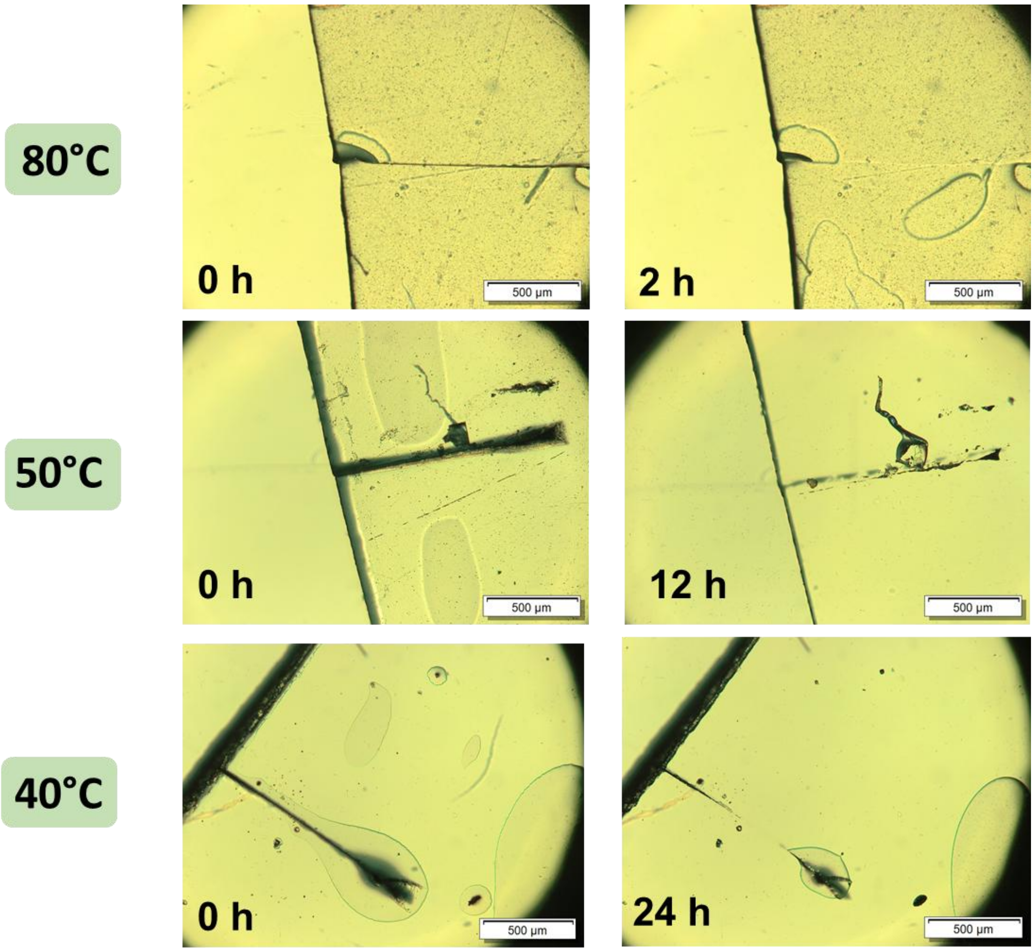

Figure S7. *Self-healing measurements for the C3 polymer membranes a time 0 (left) and at final time (right), at 80ºC (top), 50ºC (middle) and 40ºC (bottom).*

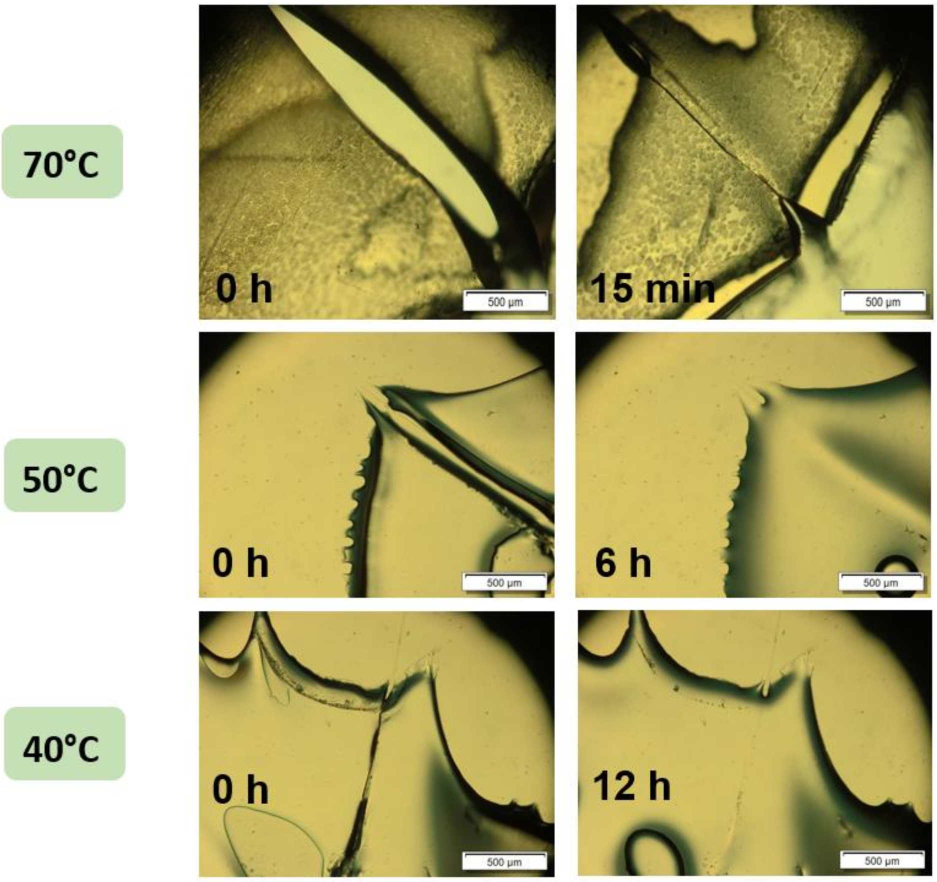

Figure S8. *Self-healing images for C4 membranes at 70ºC (top), 50ºC (middle) and 40ºC (bottom). The images show the initial time (left) and the final time (right).*

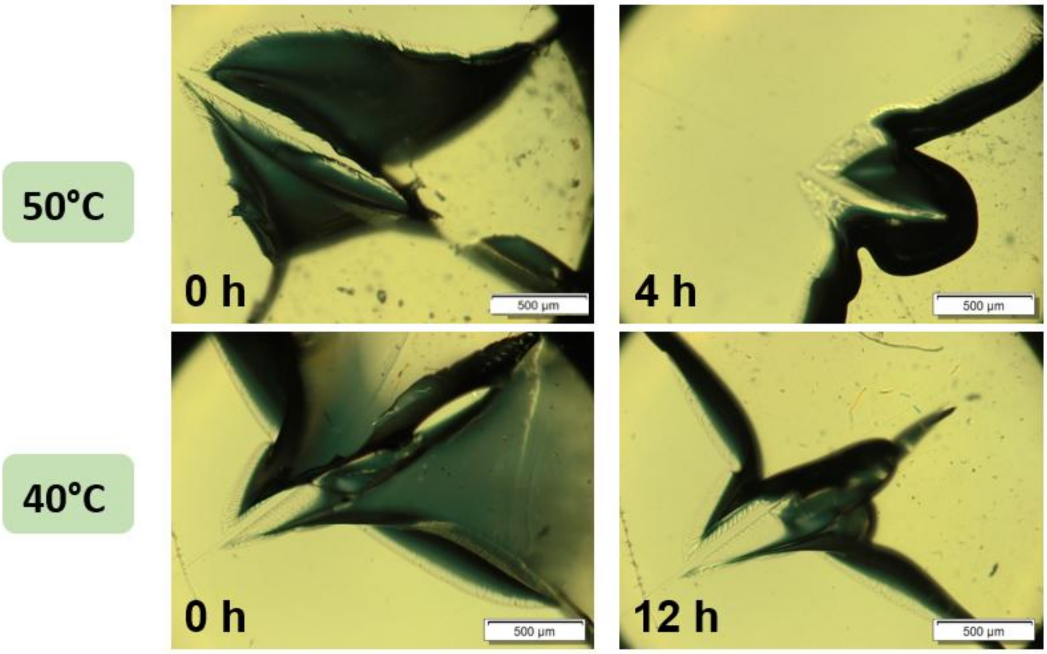

Figure S9. *Self-healing measurements for the C5 membranes.*

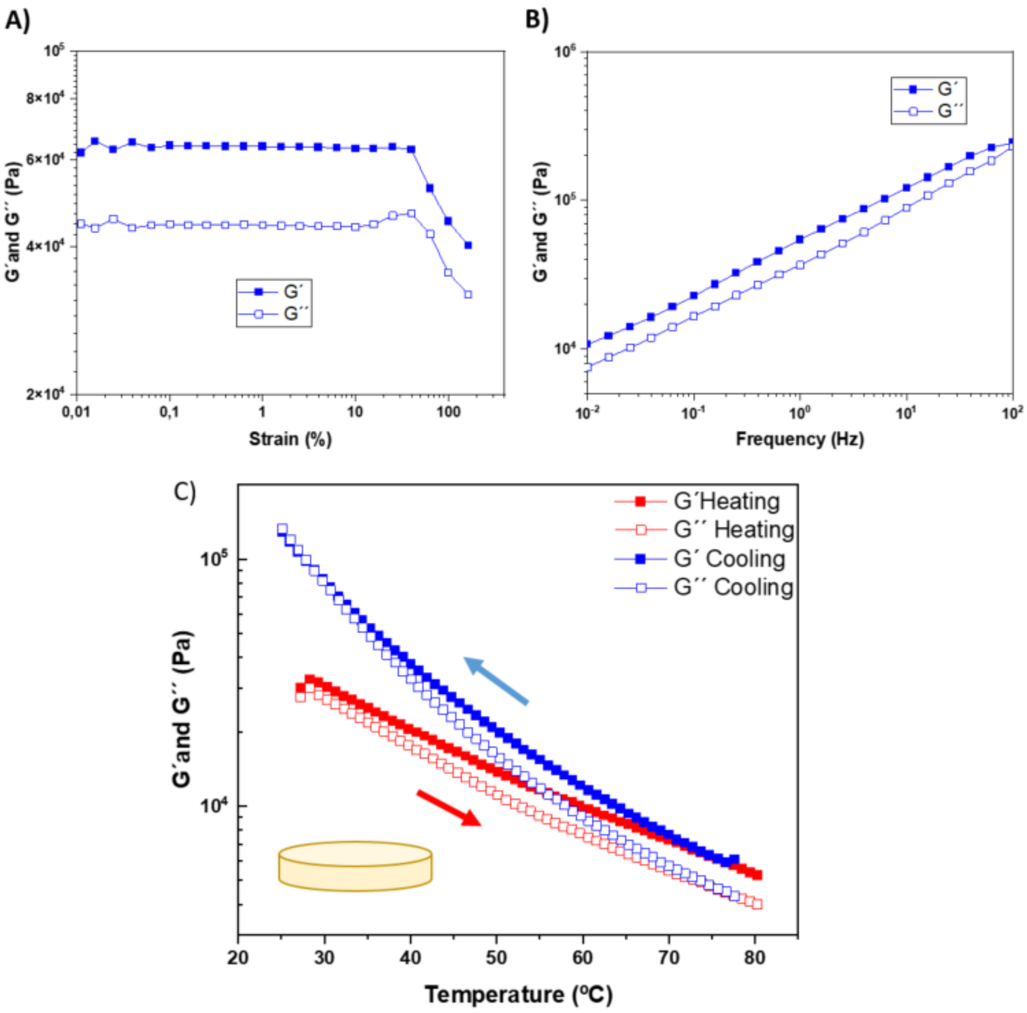

Figure S10. *Rheological experiments for C3 membranes. A) Strain test. B) Frequency test. C) Temperature Treatment, heating-cooling cycle.*

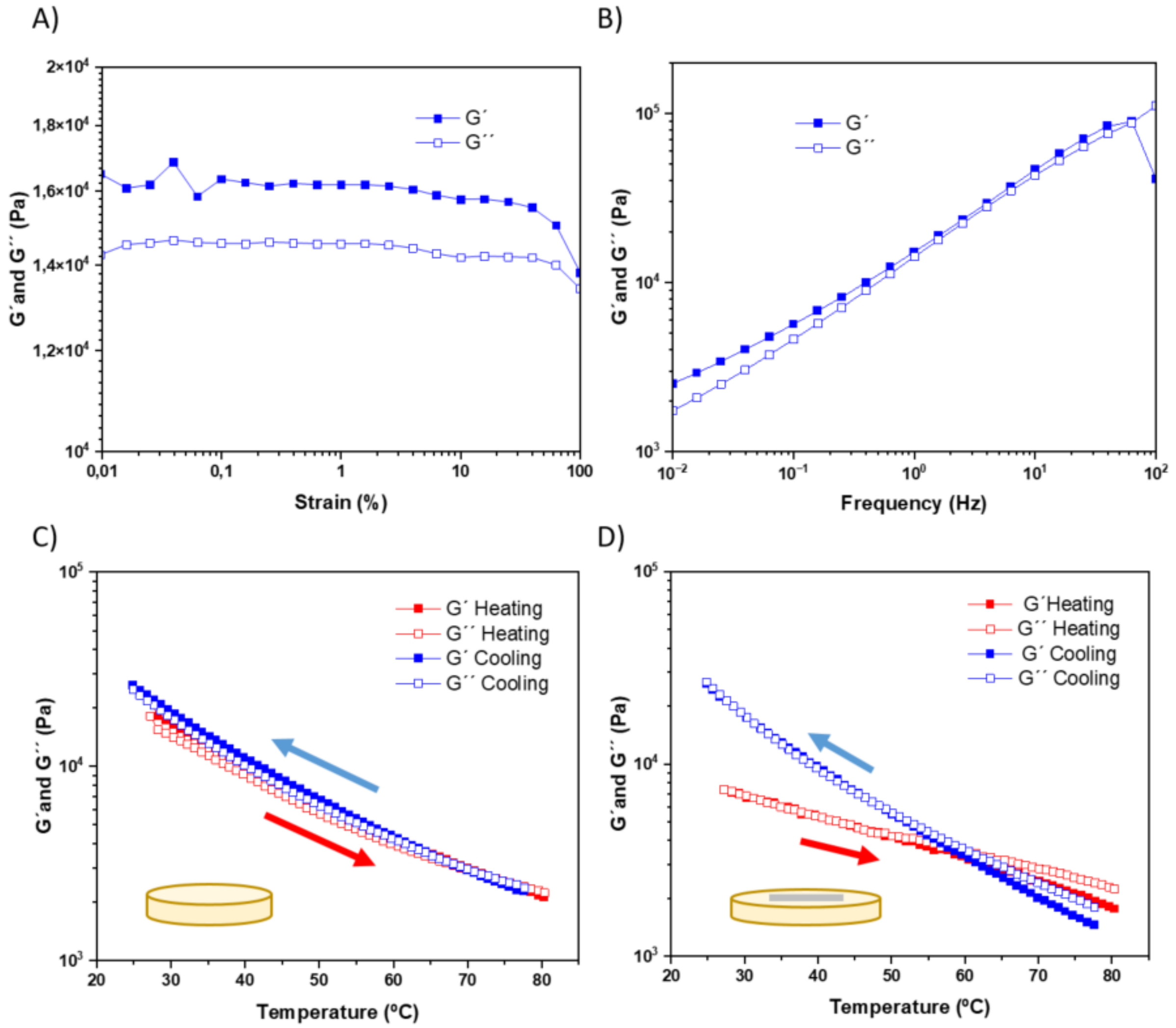

Figure S11. Rheological experiments for C4 membranes. A) Strain test. B) Frequency test. C) Temperature Treatment, heating-cooling cycle. D) Study of self-healing behavior of C4 copolymer performing rheological experiments.

Table S3. Calculated Activation Energy values in kJ/mol.

Sample	C1	C2	C3	C4	C5	Poly(PEGMEMA)
Ea (kJ/mol)	53.83	47.58	47.57	39.61	32.70	31.95

Table S4. Adhessive Stress, adhessive strain and adhession energy values taken from probe tack tests for C3, C4 and C5 copolymers.

Material	Adhessive Stress (MPa)	Adhessive Strain (%)	Adhession Energy (J/m^2)
C3	1.12	274	126.9 ± 14.9
C4	0.18	718	246.9 ± 35.6
C5	0.17	4508	124.1 ± 17.4

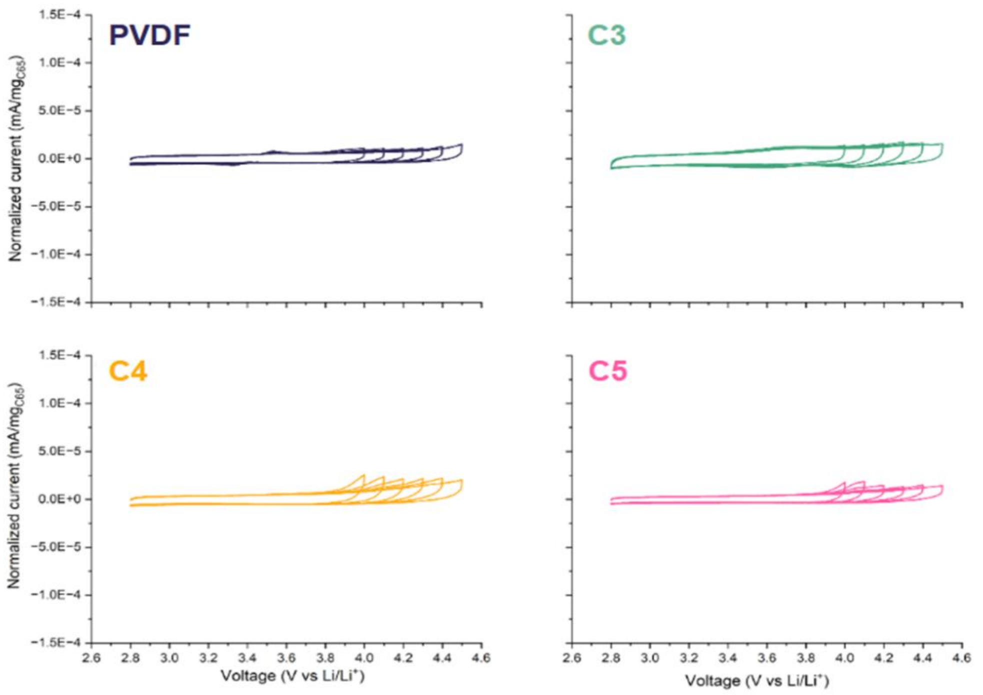

Figure S12. *Cyclic Voltammetry. Plots for C3, C4, C5 and PVDF materials in the range of 2.8-4.5V vs Li/Li*$^+$.

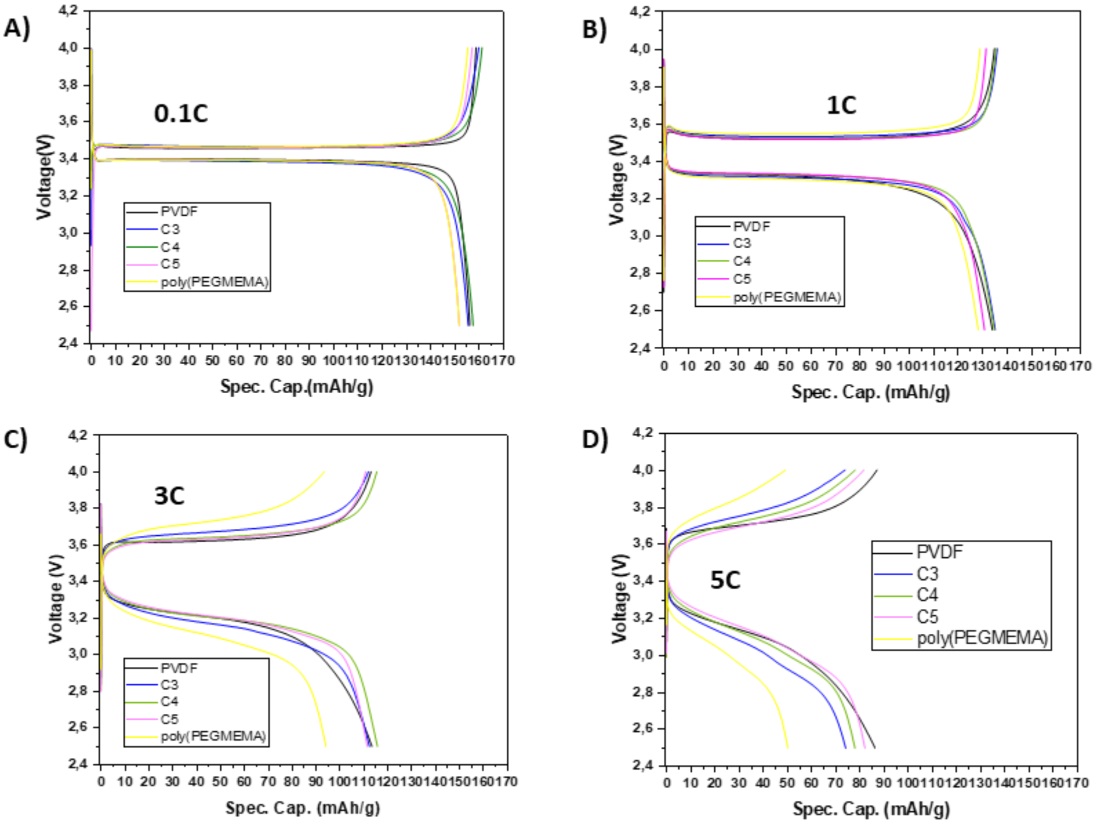

Figure S13. *Voltage profiles of PVDF, C3, C4, C5 and poly(PEGMEMA) materials at A) 0.1C, B) 1C, C) 3C and D) 5 C-rates.*
